# Cost minimizing planning of container inspection and repair in multiple facilities

**DOI:** 10.1007/s00291-022-00699-4

**Published:** 2022-12-19

**Authors:** Mikhail Y. Kovalyov, Mikhail N. Lukashevich, Erwin Pesch

**Affiliations:** 1 United Institute of Informatics Problems, National Academy of Sciences of Belarus, Surganova, 6, 220012 Minsk, Belarus; 2 Belarusian State University, Nezavisimosti avenue, 4, 220030 Minsk, Belarus; 3 Institute of Information Systems, Faculty III, University of Siegen, 57068 Siegen, Germany

**Keywords:** Optimal planning, Container repair, Min-cost multi-commodity flow, Capacitated economic lot-sizing, Integer linear programming

## Abstract

A problem of optimal mid-term or long-term planning of inspection and repair of freight containers in multiple facilities is introduced and investigated. The containers are of different types and quality levels, which define their repair costs and workforce requirements. The objective function includes the total holding, inspection, repair, transportation and rejection costs. We propose a deterministic, time-dependent, integer linear min-cost multi-commodity network-flow formulation. The problem is shown to be polynomially solvable if there is a single facility, a single time period and all the containers are repairable and have to be repaired. It is shown to be NP-hard for three important special cases. The computational results of our experiments on randomly generated instances based on real data show that instances of sizes 3 facilities, 4 container types and up to 9 container quality levels can be solved with CPLEX in 5 minutes on a conventional PC, even for 30 periods, with an optimality gap of less than 3%. This is sufficient for medium-term or weekly planning or for short-term recovery planning. However, there are instances of the same magnitude, but with 360 periods of a considerably longer planning horizon, for which an optimality gap of 28% remained even after 10 hours of CPLEX computation.

## Introduction

Container port logistics is an important sector of world economy. In 2021, global container shipping market reached approximately 849 million twenty-foot equivalent units, which is an increase of 7% compared with 2020, when the Coronavirus (COVID-19) pandemic caused cancellations of many container shipping contracts (https://www.statista.com/statistics/913398/container-throughput-worldwide/). However, as of February 2022, some 11.6% of the total container ship capacity still was not utilized. In April 2022, the biggest worldwide container port of Shanghai was paralyzed by a strict COVID lockdown. One of the core problems in container transport is the availability of empty containers exactly where they are needed, namely at suppliers of products. This increased demand for empty containers and the associated transportation is caused by our increased demand for goods produced around the world. European and American ports have a high surplus of empty containers. At the same time, Asian ports face a significant shortage, see (Kuzmicz and Pesch 2019) for empty container balancing between export and import dominant regions. In this situation, extending container lifetime and their efficient usage are important.

Container damage interrupts the flow of goods in maritime logistics, but it is inevitable and can happen at any stage of container shipping. Therefore, container inspection and repair shops exist in all major ports. Our study is motivated by the need of cost efficient planning of such shops. The empty containers, some of which can be damaged, appear in a specific place because of their planned relocation in a supply chain, or due to the completion of a shipping contract. The repaired containers are used to satisfy the demand for empty containers. This demand exists in each container transshipping point such as a port. A formal definition of the problem studied in this paper is given below.

There are empty containers of *n* types arriving to one of several repair facilities of a logistics company in the planning horizon of *T* unit-time periods. We denote this facility as 0, other facilities as $1,\ldots ,F$, and number the time periods by $t=1,\ldots ,T$. Due to limited storage, transportation, inspection and repair capacities, some of the arriving containers can be rejected.

A container that arrived at facility 0 undergoes a quality inspection at this facility and possibly needs to be repaired at this or any other facility. If a repair is performed at a facility $f\ge 1$, then the container is transported there by a vehicle. Any vehicle can carry independent of the type the same number of containers - either one or two of any type. Good quality containers are used to satisfy the given demands of the clients specified for each time period. The demanded good quality containers are collected at the time of the demand satisfaction by the clients at their expense. The remaining good quality containers are kept to satisfy the future demand. If the inspection determines that the quality of a container is not good but appropriate for repair, then it is repaired at the facility within the time period when it is brought there. Any container can have one of $q=0,1,\ldots ,Q+1$ quality levels including the good (“as new”) quality level $q=0$.

A container of any quality level but the quality level $Q+1$ can be repaired. If the inspection determines that the quality level is $Q+1$, then the corresponding container is discarded at no cost and cannot be used to satisfy any demand. We may assume that any repaired container has the required good quality. Our discussion with a container logistics company revealed that their main objective concerning scheduling inspection and repair of containers is minimizing the total costs, provided that customer demands are met and workforce, storage and transportation capacities are not exceeded. The costs include rejection, inspection, repair, inventory and transportation costs. A variation of the problem where containers must not be rejected is also interesting. Currently, the company does not optimize the costs or does it intuitively and experience-based. Their major objective is the demand satisfaction. However, the demand can be acceptably satisfied at different resource costs. Therefore, we aim to minimize a cost combination, in which the demand satisfaction can be prioritized.

Let us explain the need to consider values of *F*, *n*, *Q* and *T* greater than one. Multiple repair facilities are often used in practice because space at the container terminal is limited and only a small inspection and repair shop can be placed close to the empty container arrival point. More repair shops can exist in the terminal vicinity to satisfy larger repair demand. Multiple container types exist due to their natural multi-type characteristics such as size (20-foot or 40-foot), floor (metal or wooden), top (hard or open) and cooling requirements for maintaining cargo temperature (refrigerated container (reefers) or non-refrigerated). Inspecting and repairing a 40-foot container usually takes more time than a 20-foot one, and inspecting and repairing a reefer requires an inspection of the motor and the container itself. There exists a nomenclature of container damages, and damages are classified into major groups corresponding to the container quality levels. Damages of the same group have similar workforce requirements and repair costs. Since essentially different numbers of containers arrive in different time periods and container demands change over time, a time decomposition approach will unlikely be efficient in solving the studied problem.

In the next section, a review of relevant literature is provided. In Sect. 3, we describe a min-cost multi-commodity network flow formulation of the studied problem, which can be classified as an *Integer Linear Programming (ILP)* problem. Section 4 establishes a borderline between easy and hard cases of the studied problem. An *O*(*n*) time algorithm is presented for the case $F=0$, $T=1$, all containers are repairable and container rejection is not allowed. When there exist non-repairable containers of each type, or one of the parameters $F=0$ or $T=1$ is increased by one, the problem becomes computationally difficult. Section 5 presents computer experiments with CPLEX for the ILP problem. The paper completes with a short summary of the results.

## Literature review

We are not aware of any academic literature on planning container inspection and repair operations. However, our problem has some common features with earlier investigated problems. In particular, the problem falls into the categories of inventory management in reverse logistics, planning work and rework processes and lifetime inspection and maintenance planning for deteriorating engineering systems. Reverse logistics deals with the reuse processes in supply chains, see (Fleischmann et al. 1997), (de Brito and Dekker 2004), (Kim and Glock 2014), (Cobb 2016), (Berling and Sonntag 2022). In a closed-loop supply chain, demands are satisfied by new and repaired products, which leads to a complex problem of planning the original production and the repair. Our problem is simpler. It does not consider flows of new and repaired containers separately as well as constraints on these flows and complex relations between the participants of the supply chain.

The literature on planning work and rework processes usually assumes that these two types of processes are performed on the same equipment, and the objective is to minimize the total cost of switching between work and rework, holding-backlogging cost, rework cost and defective product disposal cost, see (Inderfurth et al. 2006, 2007). Comparing with these studies, our problem is again easier because production of new containers is not considered. However, our problem considers several specific conditions such as different container types and quality levels, workforce, storage and transportation capacities and costs of the operations.

A number of publications deal with the lifetime inspection and maintenance planning for deteriorating engineering systems. For example, (Bismut and Straub 2021) propose a heuristic-based adaptive planning method for inspection and maintenance of large stochastically deteriorating structures during their service life. (Morato et al. 2022) combine dynamic Bayesian networks with partially observable Markov decision processes for optimal inspection and maintenance planning of civil and maritime engineering systems, which are exposed to fatigue and corrosion throughout their operational life. Contrary to these studies, we do not consider long-term timing decisions – the containers are inspected and repaired soon after their arrival to the port.

Several studies exist in which container inspection or repair is mentioned as a logistics system element, but container inspection and repair planning is not part of the decision. (Weele and Ramirez-Marquez 2010) propose an optimization technique for constructing an inspection strategy that delivers a given detection rate for contraband-containing containers at the lowest possible cost.(Bernat et al. 2016) present stochastic review policies that incorporate allocation schemes for empty containers, their maintenance and repair options, as well as street turns. (Hjortnaes et al. 2017) propose a multi-commodity model to optimize empty container repositioning strategies, which makes an explicit distinction between flows of non-damaged and damaged containers. (Hosseini and Sahlin 2019) develop a multi-period optimization model to dispatch empty containers of multiple types and various conditions (dirty and clean) between terminals, depots and cleaning stations. Our results may complement these studies by adding consideration of inspection and/or repair resources and costs to the respective models.

Our solution approach is to formulate the studied problem as a min-cost multi-commodity network flow problem. Problems of this type are studied by (Ahuja et al. 1993), (Karzanov 1994), and recently by (Grande et al. 2018) and (Khodayifar 2021), to name a few. The typical application is to find the best delivery plan of several products from the manufacturing sites to the warehouses though a given road network with arc capacities and costs. The common features of these problems and our problem are several incoming flows that go through a space-time network, the flow conservation constraints and the total flow cost to be minimized. The resource constraints, which are crucial in our problem, are rarely considered in the network flow problems. The studied problem can be also classified as a multi-item dynamic economic lot-sizing problem, which is to find production quantities of a manufacturing line over a discrete time horizon such that the production capacity is not exceeded and the total setup, production and holding/backlogging cost is minimized. Studies of single-item problems of this kind were initiated by (Wagner and Whitin 1958). Multi-item problems, resource constrained problems and problems with ecological/re-manufacturing considerations of this kind have been studied by (Kao 1979), (Chubanov et al. 2008), (Li et al. 2012), (Kim and Lee 2013), (Akbalik et al. 2015), (Kang et al. 2018), (Wu et al. 2018), (Absi et al. 2018), (Cunha et al. 2019), (Altendorfer 2019), (Kilic and van den Heuvel 2019), (Beck and Glock 2020), among others. The specific combination of the production environment, resource constraints and costs in our problem has not been studied before.

## Detailed problem formulation

We first introduce and explain input data for the studied problem in Table 1.

**Table 1:** Input data

Notation	Description
*n*	Number of container types
*Q*	Quality levels. Level 0 corresponds to perfect quality and level $Q+1$ to unacceptable level
*F*	Inspection is done on facility 0 and repair is done on facilities $0,1,\ldots ,F$
*T*	Number of time periods in the planning horizon
${c}_{j}^{({{rej}})}$	Cost of rejection of one container of type *j*, $j=1,\ldots ,n$
$c^{(ins)}_j$	Cost of inspection of one container of type *j* and any quality level at any facility, $j=1,\ldots ,n$
$$ c_{{jq}}^{{({{rep}})}}$$	Cost of repair of one container of type *j* and quality level *q* at any facility, $$j=1,\ldots ,n$$, $$q=0,1,\ldots ,Q$$, $$c^{(rep)}_{j0}=0$$ (for good quality containers)
$c_{j}^{{({{rep}})}} = \frac{{\sum\limits_{{q = 0}}^{Q} {c_{{{\text{jq}}}}^{{({{rep}})}} } p_{{{\text{jq}}}} }}{{100}}$	Expected cost of repair of one container of type *j* and any quality level but quality level $Q+1$ at any facility, $j=1,\ldots ,n$
$c_{f}^{{({{tra}})}}$	Cost of transportation of one container of any type from facility 0 to facility *f*, $f=1,\ldots ,F$
$c_{f}^{{({{hol}})}}$	Holding cost of one container of any type at facility *f* when passing from any time period *t* to time period $t+1$, $t=0,1,\ldots ,T$, $f=0,1,\ldots ,F$
$d_{{{\text{jt}}}}$	Demand of containers of type *j* which is the number of good quality containers of type *j* that should be available for clients by the end of time period *t*, $j=1,\ldots ,n$, $t=1,\ldots ,T$. This demand can be satisfied by containers repaired in time periods $1,\ldots ,t$, and it cannot be satisfied by those repaired in the later time periods
$g_{{{\text{jf}}}}$	Initial inventory of good quality containers of type *j* at facility *f* at the beginning of time period 1, $j=1,\ldots ,n$, $f=0,1,\ldots ,F$
$k_{{{\text{jt}}}}$	Number of containers of type *j* arriving to facility 0 at the beginning of time period *t*, $j=1,\ldots ,n$, $t=1,\ldots ,T$
*m*	Transportation capacity, which is the total number of containers of any type that can be moved from facility 0 to all other facilities in any single time period. If the set *M* of vehicles, their container capacities $cap_v$ and numbers of journeys $num_v$ in any single time period are known, then $m=\sum _{v\in M}cap_vnum_v$
$\frac{p_{jq}}{100}$	Probability that the quality level of any single non-inspected container of type *j* is *q*, $p_{jq}\in \{0,1,\ldots ,100\}$, $j=1,\ldots ,n$, $q=0,1,\ldots ,Q+1$, $\sum _{q=0}^{Q+1} p_{jq}=100$. It can be a historical percentage of containers of quality level *q* among all the inspected containers of type *j*. It is restrictively assumed that the number of containers of quality level *q* among any *x* non-inspected containers of type *j* is equal to $\frac{p_{jq}x}{100}$ for any *x*. (Inderfurth et al. 2006),(Inderfurth et al. 2007) point out that it is often the case in practice that statistical observations provide information about the usual percentage of defective items
$r_{jq}$	Number of working-hours required to repair one container of type *j* of quality level $q\ne Q+1$ at any facility, $j=1,\ldots ,n$, $q=0,1,\ldots ,Q$, $r_{j0}:=0$ (for good quality containers)
$r_j=\frac{\sum _{q=0}^Qr_{jq}p_{jq}}{100}$	Expected number of working-hours required to repair one container of type *j* of any quality level but quality level $Q+1$ at any facility, $j=1,\ldots ,n$
$s_j$	Number of working-hours required to inspect one container of type *j* at any facility, $j=1,\ldots ,n$
$U_f$	Upper bound on working-hours available for repair work in any time period at facility *f*, $f=0,1,\ldots ,F$
$u_{jf}$	Initial inventory of non-inspected containers of type *j* at facility *f* at the beginning of time period 1, $j=1,\ldots ,n$, $f=0,1,\ldots ,F$
$V_f$	Upper bound on working-hours available for inspection in any time period at facility *f*, $f=0,1,\ldots ,F$
$W_f$	Storage capacity of facility *f*, which is the number of containers of all types that can be simultaneously kept at *f*, $f=0,1,\ldots ,F$

All the input parameters are assumed to be nonnegative integer numbers. In reality, the numbers of arriving containers, their quality and, to some extent, container demands are uncertain. We assume that their values are given by experts on the basis of previous experience and statistical data from the same or a similar company. The income of the container repair business is assumed to be fixed because all the demands must be satisfied. Therefore, the profit is maximized by minimizing the costs.

**Fig. 1:**
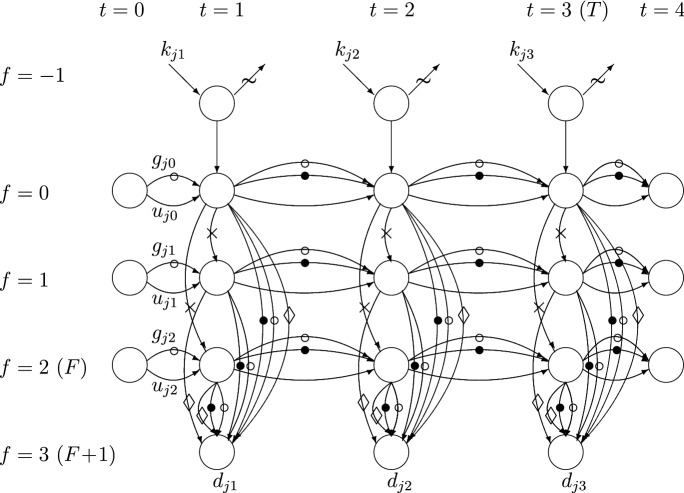
Network for the min-cost multi-commodity flow formulation, $F=2$, $T=3$.

The cost minimizing problem admits a constrained min-cost multi-commodity network flow formulation which is illustrated in Fig. 1. The corresponding network is denoted as $G=(O,A)$ where *O* and *A* define the sets of nodes and arcs, respectively, in the network. It is assumed that there are *n* container flows of types $j=1,\ldots ,n$ along each arc $a\in A$. Nodes are denoted by pairs (*f*, *t*), $f=-1,0,1,\ldots ,F+1$, $t=0,1,\ldots ,T+1$, where $f=-1,F+1$ denote artificial facilities corresponding to the arrival and rejection stage ($f=-1$), and departure of good quality and non-repairable containers from the system ($f=F+1$). An arc connecting node *o* to node *q* is denoted by a triple $(o,q,\cdot )$, where symbol $\cdot \in \{\thicksim ,-,\circ ,\bullet ,\times ,\lozenge \}$ is used to distinguish the type of container flow on parallel arcs. Nodes corresponding to the container arrival and removal of rejected containers are not shown in Fig. 1. Arcs corresponding to the flow of rejected containers are marked with symbol $\thicksim$. Nodes and arcs of the other types are explained below.

For modeling purposes it is convenient to introduce the following notation.

- $O_0$ – set of nodes each of which has at least one predecessor and at least one successor.
- $P_v$ – set of *ingoing arcs* of the node $v\in O_0$.
- $S_v$ – set of *outgoing arcs* of the node $v\in O_0$.
- $A^-$ – set of arcs corresponding to the flow of arriving and accepted containers via facility $-1$ and to the flow of non-inspected and non-repaired containers over time at the same facility $f\in \{0,1,\ldots ,F\}$. These are the unmarked arcs in Fig. 1.
- $A^\circ$ – set of arcs $((f,t),(f,t+1),\circ )$ and $((f,t),(F+1,t),\circ )$ corresponding to the good quality containers kept at facility *f* in the time period from *t* to $t+1$ and that are carried from facility *f* to facility $F+1$ in the same time period *t*, respectively. These containers were quality checked at time $t-1$ or earlier and are therefore known to be of good quality when they arrive to facility *f* at *t*. These arcs are marked with symbol “$\circ$” in Fig. 1.
- $A^\bullet _{ft}$ – set of arcs $((f,t),(f,t+1),\bullet )$ and $((f,t),(F+1,t),\bullet )$ corresponding to the flow of good quality containers, which were inspected and possibly repaired at facility *f* at time *t*. These arcs are marked with symbol “$\bullet$” in Fig. 1.
- $$A^\bullet =\cup _{f=0}^F\cup _{t=1}^T A^\bullet _{ft}$$.
- $A^\times _{ft}$ – set of arcs corresponding to the transportation of non-inspected and non-repaired containers from facility 0 to facility $f\in \{1,\ldots ,F\}$ in time period *t*. These arcs are marked with symbol “$\times$” in Fig. 1.
- $$A^\times =\cup _{f=1}^F\cup _{t=1}^T A^\times _{ft}$$.
- $A^\lozenge$ – set of arcs corresponding to the flow of non-repairable containers among containers inspected for quality. These arcs are marked with symbol “$\lozenge$” in Fig. 1.
- $A^\thicksim$ – set of arcs corresponding to the flow of rejected containers. These arcs are marked with symbol “$\thicksim$” in Fig. 1.

The constrained min-cost multi-commodity network flow problem is denoted as CMCF (Constrained Min-Cost Flow). In the algebraic form, it can be given as follows. Introduce nonnegative integer variables $x_{ja}$ which represent the amount of flow (number of containers) of type *j* along the arc *a*, $j=1,\ldots ,n$, $a\in A$. Denote by *x* the matrix of variables $x_{ja}$ and denote by $\mathbb {N}^{n,|A|}_0$ the set of all such nonnegative integer matrices.

**Problem** CMCF :

$$ \min _{x} \;\;\sum\limits_{{j = 1}}^{n} ( {\text{Rej}}_{j} (x) + Hol_{j} (x) + IR_{j} (x) + {\text{Tra}}_{j} (x)), $$

where

$$\begin{aligned} Rej_j(x)=c^{(rej)}_j\sum _{t=1}^Tx_{j((-1,t),(-1,t),\thicksim )} \end{aligned}$$

are the total rejection costs of containers of type *j*,

$$\begin{aligned} Hol_j(x)=\sum _{t=1}^T\sum _{f=0}^F c^{(hol)}_f(x_{j((f,t),(f,t+1),-)}+x_{j((f,t),(f,t+1),\circ )}+x_{j((f,t),(f,t+1),\bullet )}) \end{aligned}$$

are the total holding costs of containers of type *j* at all the facilities,

$$\begin{aligned} IR_j(x)=(c^{(ins)}_j+c^{(rep)}_j)\sum _{a\in A^\bullet } x_{ja}+c^{(ins)}_j\sum _{a\in A^\lozenge } x_{ja} \end{aligned}$$

are the total inspection and repair costs of containers of type *j*, and

$$\begin{aligned} Tra_j(x)=\sum _{t=1}^T\sum _{f=1}^Fc^{(tra)}_f\sum _{a\in A^\times _{ft}} x_{ja} \end{aligned}$$

are the total transportation costs of containers of type *j*, subject to

1 $$\begin{aligned}&x_{j((f,0),(f,1),\circ ))}=g_{jf},\ j=1,\ldots ,n,\ f=0,1,\ldots ,F, \end{aligned}$$

2 $$\begin{aligned}&x_{j((f,0),(f,1),-))}=u_{jf},\ j=1,\ldots ,n,\ f=0,1,\ldots ,F, \end{aligned}$$

3 $$\begin{aligned}&x_{j((-1,t),(0,t),-))}+x_{j((-1,t),(-1,t),\thicksim )}=k_{jt},\ j=1,\ldots ,n,\ t=1,\ldots ,T, \end{aligned}$$

4 $$\begin{aligned}&\sum _{a\in S_v\cap A^\lozenge } x_{ja}=\Big \lfloor \frac{p_{j,Q+1}}{100}\sum _{a\in S_v\cap (A^\lozenge \cup A^\bullet )} x_{ja}\Big \rfloor ,\ v\in O_0,\ j=1,\ldots ,n, \end{aligned}$$

5 $$\begin{aligned}&\sum _{a\in P_v\cap (A^-\cup A^\times )} x_{ja}=\sum _{a\in S_v\backslash A^\circ }x_{ja},\ v\in O_0,\ j=1,\ldots ,n, \end{aligned}$$

6 $$\begin{aligned}&\sum _{a\in P_v\cap (A^\circ \cup A^\bullet )} x_{ja}=\sum _{a\in S_v\cap A^\circ } x_{ja},\ v\in O_0,\ j=1,\ldots ,n, \end{aligned}$$

7 $$\begin{aligned}&\sum _{a\in P_v\backslash A^\lozenge } x_{ja}=d_{jt},\ v=(F+1,t),\ j=1,\ldots ,n,\ t=1,\ldots ,T, \end{aligned}$$

8 $$\begin{aligned}&\sum _{j=1}^n\sum _{f=1}^F\sum _{a\in A^\times _{ft}} x_{ja}\le m,\ t=1,\ldots ,T, \end{aligned}$$

9 $$\begin{aligned}&\sum _{j=1}^n s_j \sum _{a\in S_{(f,t)}\cap (A^\lozenge \cup A^\bullet )} x_{ja}\le V_f,\ t=1,\ldots ,T,\ f=0,1,\ldots ,F, \end{aligned}$$

10 $$\begin{aligned}&\sum _{j=1}^n r_j \sum _{a\in A^\bullet _{ft}} x_{ja}\le U_f,\ t=1,\ldots ,T,\ f=0,1,\ldots ,F, \end{aligned}$$

11 $$\begin{aligned}&\sum _{j=1}^n (x_{j((f,t),(f,t+1),-)}+x_{j((f,t),(f,t+1),\circ )}+x_{j((f,t),(f,t+1),\bullet )})\\& \quad \le W_f,\ t=1,\ldots ,T,\ f=0,1,\ldots ,F, \end{aligned}$$

12 $$\begin{aligned}&x\in \mathbb {N}^{n,|A|}_0. \end{aligned}$$

Constraints (1)–(3) initialize the input flow variables and set them to the prescribed values. Constraints (4) define the quantity of non-repairable containers. The set $S_v\cap (A^\lozenge \cup A^\bullet )$ in (4), where $v=(f,t)$, corresponds to the flow of containers which were uninspected at facility *f* at the beginning of time period *t*. Then, they were inspected and repaired there, and the flow was divided into the flow of non-repairable containers marked $\lozenge$, and two flows of good quality containers marked $\bullet$, namely, one flow to satisfy the demand at time period *t* and another flow to satisfy the demands in later time periods. Constraints (5) and (6) are the flow conservation constraints. Note that, due to (5) and the rounding down operation in (4), the fraction of the non-repairable containers of type *j* among all containers of this type inspected at facility *f* in time period *t* can be less than the assumed fraction $\frac{p_{j,Q+1}}{100}$. Constraints (7) are the demand satisfaction constraints. Constraints (8) are the transportation capacity constraints. Constraints (9) and  (10) are the resource constraints linked with the available working-hours for the inspection work and the repair work, respectively. Constraints (11) are the storage capacity constraints. Constraints (12) state that the flow values are nonnegative integer numbers.

By substituting (4) with the equivalent relations

$$\begin{aligned} p_{j,Q+1}\sum _{a\in S_v\cap A^\bullet } x_{ja}-99\le (100-p_{j,Q+1})\sum _{a\in S_v\cap A^\lozenge } x_{ja},\ v\in O_0,\ j=1,\ldots ,n, \end{aligned}$$

$$\begin{aligned} (100-p_{j,Q+1})\sum _{a\in S_v\cap A^\lozenge } x_{ja}\le p_{j,Q+1}\sum _{a\in S_v\cap A^\bullet } x_{ja},\ v\in O_0,\ j=1,\ldots ,n, \end{aligned}$$

the problem CMCF becomes an ILP problem. A relaxation of CMCF, in which variables $x_{ja}$ are rational, is polynomially solvable. We denote this relaxed problem as CMCF-R. The optimal objective value of CMCF-R can be used as a lower bound for the optimal value of CMCF.

## Computational complexity

We demonstrate in this section that the problem CMCF with no rejection is solvable in *O*(*n*) time if $F=0$, $T=1$ and all containers are repairable, and it is NP-hard if there exist non-repairable containers of each type or one of the parameters *F* and *T* is increased by one. We begin with the polynomially solvable case. Denote this case as CMCF$(F=0,T=1,c^{(rej)}_j=\infty ,p_{j,Q+1}=0)$. The case $F=0$ of a single facility often happens in reality. The case $T=1$ of one time period can be employed in a rolling horizon solution approach when part of the decision is fixed and another part is to be adjusted to the real-life changes. The case $p_{j,Q+1}=0$, $j=1,\ldots ,n$, reflects the situation when all the containers are repairable. Let us show that CMCF$(F=0,T=1,c^{(rej)}_j=\infty ,p_{j,Q+1}=0)$ can be solved in *O*(*n*) time.

### Theorem 1

The problem CMCF$(F=0,T=1,c^{(rej)}_j=\infty ,p_{j,Q+1}=0)$ has no solution if and only if any of the following inequalities is satisfied:

13 $$\begin{aligned}&g_{j0}+u_{j0}+k_{j1}<d_{j1},\ \ j=1,\ldots ,n, \end{aligned}$$

14 $$\begin{aligned}&\sum _{j=1}^n s_j(d_{j1}-\min \{g_{j0},d_{j1}\})>V_0, \end{aligned}$$

15 $$\begin{aligned}&\sum _{j=1}^nr_j (d_{j1}-\min \{g_{j0},d_{j1}\})>U_0, \end{aligned}$$

16 $$\begin{aligned}&\sum _{j=1}^n(g_{j0}+u_{j0}+k_{j1}-d_{j1})>W_0. \end{aligned}$$

If relations reversed to (13)–(16) are satisfied, then the following solution is optimal: $x_{j((0,1),(1,1),\lozenge )}=0$, $x_{j((0,1),(1,1),\circ )}=\min \{g_{j0},d_{j1}\}$, $x_{j((0,1),(0,2),\circ )}=g_{j0}-x_{j((0,1),(1,1),\circ )}$, $x_{j((0,1),(1,1),\bullet )}=d_{j1}-x_{j((0,1),(1,1),\circ )}$, $x_{j((0,1),(0,2),\bullet )}=0$, $x_{j((0,1),(0,2),-)}=u_{j0}+k_{j1}-x_{j((0,1),(1,1),\bullet )}$, $j=1,\ldots ,n$.

### Proof

The value in the left-hand side of (13) is the number of available containers of type *j*, for all *j*. If that number does not satisfy the demand, then the problem has no solution. Further, since there are $g_{j0}$ good quality containers of type *j*, the minimum number of containers of type *j* to be inspected and repaired in order to satisfy the demand is $d_{j1}-\min \{g_{j0},d_{j1}\}$. Therefore, if any of (14) and (15) is satisfied, then the problem has no solution because of the resource shortage. The inventory of type *j* containers at the end of time period 1 is equal to their initial inventory $g_{j0}+u_{j0}+k_{j1}$ minus the demand $d_{j1}$. Therefore, if (16) is satisfied, then the problem has no solution because of the insufficient storage capacity.

Now, assume that (13)–(16) are satisfied. The problem CMCF in the statement of the theorem can be represented as follows.

$$\begin{aligned}&\min _{x}\sum _{j=1}^n[c^{(hol)}_0(g_{j0}+u_{j0}+k_{j1}-d_{j1})\\& \quad +(c^{(ins)}_j+c^{(rep)}_j)(x_{j((0,1),(0,2),\bullet )} +x_{j((0,1),(1,1),\bullet )})],\\&\hbox { subject to } \end{aligned}$$

17 $$\begin{aligned}&g_{j0}=x_{j((0,1),(0,2),\circ )}+x_{j((0,1),(1,1),\circ )},\ j=1,\ldots ,n, \end{aligned}$$

18 $$\begin{aligned}&x_{j((0,1),(1,1),\bullet )}+x_{j((0,1),(1,1),\circ )}=d_{j1},\ j=1,\ldots ,n, \end{aligned}$$

19 $$\begin{aligned}&u_{j0}+k_{j1}=x_{j((0,1),(0,2),-)}+x_{j((0,1),(0,2),\bullet )}+x_{j((0,1),(1,1),\bullet )},\ j=1,\ldots ,n, \end{aligned}$$

20 $$\begin{aligned}&\sum _{j=1}^n s_j (x_{j((0,1),(1,1),\bullet )}+x_{j((0,1),(0,2),\bullet )})\le V_0, \end{aligned}$$

21 $$\begin{aligned}&\sum _{j=1}^nr_j (x_{j((0,1),(1,1),\bullet )}+x_{j((0,1),(0,2),\bullet )})\le U_0, \end{aligned}$$

22 $$\begin{aligned}&\sum _{j=1}^n(x_{j((0,1),(0,2),-)}+x_{j((0,1),(0,2),\circ )}+x_{j((0,1),(0,2),\bullet )})\le W_0,\nonumber \\&x\in \mathbb {N}^{n,|A|}_0. \end{aligned}$$

Observe that the solution presented in the statement of the theorem satisfies the constraints (17)–(22). Furthermore, the maximal quantities of good quality containers are used to satisfy the demands, and therefore, the inspection and repair costs are minimized, which means that the presented solution is optimal. $\square$

Denote a modification of the polynomially solvable problem CMCF$(F=0,T=1,c^{(rej)}_j=\infty ,p_{j,Q+1}=0)$, in which $p_{j,Q+1}>0$, $j=1,\ldots ,n$, (there exist non-repairable containers of each type) as CMCF$(F=0,T=1,c^{(rej)}_j=\infty ,p_{j,Q+1}>0)$. We show that the modified problem is NP-hard.

### Theorem 2

The problem CMCF$(F=0,T=1,c^{(rej)}_j=\infty ,p_{j,Q+1}>0)$ is NP-hard even if $g_{j0}=u_{j0}=d_{j1}=0$, $j=1,\ldots ,n$, and all the repair and holding costs are equal to zero.

### Proof

We will use a reduction from the NP-complete problem Equal Cardinality Partition (ECP) ( (Garey and Johnson 1979)).

ECP: Given positive integer numbers $a_1,\ldots ,a_{2h}$ and *A* satisfying $\sum _{j=1}^{2h} a_j=2A$, is there a subset $Y\subset H:=\{1,\ldots ,2h\}$ such that $|Y|=h$ and $\sum _{j\in Y}a_j=A$?

For any instance of ECP, construct an instance of CMCF$(F=0,T=1,c^{(rej)}_j=\infty ,p_{j,Q+1}>0)$, in which $n=2h$, $W_0=3h$, $V_0= 4h^2A-2A$, $g_{j0}=u_{j0}=d_{j1}=c^{(rep)}_j=c^{(hol)}_f=0$, $k_{j1}=2$, $p_{j,Q+1}=50$, $c^{(ins)}_j=a_j$, $s_j=2hA-a_j$, $j=1,\ldots ,n$. Values of the other input parameters are such that the constraints they are involved in are satisfied. The case of the problem CMCF in the statement of the theorem can be represented as follows.

$$\begin{aligned} \min _{x}\sum _{j=1}^{2h} a_j(x_{j((0,1),(0,2),\bullet )}+x_{j((0,1),(1,1),\lozenge )}), \hbox { subject to } \end{aligned}$$

23 $$\begin{aligned}&x_{j((0,1),(0,2),\bullet )}+x_{j((0,1),(1,1),\lozenge )}+x_{j((0,1),(0,2),-)}=k_{j1}=2,\ j=1,\ldots ,2h, \end{aligned}$$

24 $$\begin{aligned}&x_{j((0,1),(1,1),\lozenge )}=\Big \lfloor \frac{50(x_{j((0,1),(0,2),\bullet )}+x_{j((0,1),(1,1),\lozenge )})}{100}\Big \rfloor ,\ j=1,\ldots ,2h, \end{aligned}$$

25 $$\begin{aligned}&\sum _{j=1}^{2h} (2hA-a_j)(x_{j((0,1),(0,2),\bullet )}+x_{j((0,1),(1,1),\lozenge )})\le 2(2h^2A-A), \ \end{aligned}$$

26 $$\begin{aligned}&\sum _{j=1}^{2h}(2-x_{j((0,1),(1,1),\lozenge )})\le 3h, \nonumber \\&x\in \mathbb {N}^{2h,|A|}_0. \end{aligned}$$

We show that the instance of ECP has a solution if and only if the constructed instance of CMCF$(F=0,T=1,c^{(rej)}_j=\infty ,p_{j,Q+1}>0)$ has a feasible solution with the total cost not exceeding 2*A*. Note that the objective function only contains inspection costs of $a_j$. Assume that the instance of CMCF$(F=0,T=1,c^{(rej)}_j=\infty ,p_{j,Q+1}>0)$ has a solution *x* with total costs of at most 2*A*. Due to the constraints (23) and (24), only the following pairs of variable values are feasible for any fixed *j*: (a) $x_{j((0,1),(0,2),\bullet )}=1$, $x_{j((0,1),(1,1),\lozenge )}=1$, (b) $x_{j((0,1),(0,2),\bullet )}=1$, $x_{j((0,1),(1,1),\lozenge )}=0$, and (c) $x_{j((0,1),(0,2),\bullet )}=0$, $x_{j((0,1),(1,1),\lozenge )}=0$. Furthermore, if $x_{j((0,1),(1,1),\lozenge )}=0$, then there exists an optimal solution such that $x_{j((0,1),(0,2),\bullet )}=0$ for the same *j*. Therefore, we may assume that any of the following two cases is realized for each *j*: (a) $x_{j((0,1),(0,2),\bullet )}=1$, $x_{j((0,1),(1,1),\lozenge )}=1$, or (c) $x_{j((0,1),(0,2),\bullet )}=0$, $x_{j((0,1),(1,1),\lozenge )}=0$.

Introduce set $Y=\{j\mid \hbox { for which case (a) is realized, } j\in H\}$. It follows from (26) that $|Y|\ge h$. We must have

$$\begin{aligned} \sum _{j=1}^{2h} a_j(x_{j((0,1),(0,2),\bullet )}+x_{j((0,1),(1,1),\lozenge )})= 2\sum _{j\in Y} a_j\le 2A \hbox { and } \end{aligned}$$

$$\begin{aligned} 2(2h^2A-A) &\ge \sum _{j=1}^{2h} (2hA-a_j)(x_{j((0,1),(0,2),\bullet )}+x_{j((0,1),(1,1),\lozenge )})\\&=4hA|Y|-2\sum _{j\in Y}a_j\ge 4h^2A-2A. \end{aligned}$$

It follows that $|Y|=h$ and $\sum _{j\in Y}a_j=A$. Hence, *Y* is a solution of the instance of ECP.

Let set *Y* be a solution of the instance of ECP. Construct a solution *x* of the instance of CMCF$(F=0,T=1,c^{(rej)}_j=\infty ,p_{j,Q+1}>0)$ such that $j\in Y$ implies case (a) and $j\in H\backslash Y$ implies case (c). It can easily be verified that *x* is feasible and the total cost is equal to 2*A*, as it is required. $\square$

We further show that increasing $F=0$ or $T=1$ in the pair $(F=0,T=1)$ by one unit makes the polynomially solvable problem CMCF$(F=0,T=1,c^{(rej)}_j=\infty ,p_{j,Q+1}=0)$ computationally difficult even if all the costs are equal to zero.

### Theorem 3

The problem CMCF$(F=1,T=1,c^{(rej)}_j=\infty ,p_{j,Q+1}=0)$ is NP-hard even if all the costs are equal to zero.

### Proof

We will use a reduction from the NP-complete problem Partition ( (Garey and Johnson 1979)).

Partition: Given positive integer numbers $a_1,\ldots ,a_l$, is there a subset $Y\subset L:=\{1,\ldots ,l\}$ such that $\sum _{j\in Y}a_j=\sum _{j\in L\backslash Y}a_j$?

Denote $A=\sum _{j=1}^l a_j/2$. For any instance of Partition, construct instance of CMCF$(F=1,T=1,c^{(rej)}_j=\infty ,p_{j,Q+1}=0)$, in which $n=l$, $U_0=U_1=A$, $k_{j1}=1$, $g_{j0}=g_{j1}=u_{j0}=u_{j1}=0$, $d_{j1}=1$ (one container of each type *j* must be repaired either on facility 0 or on facility 1), $p_{j,Q+1}=0$ (all containers are repairable), and repair workforce requirements are $r_j=a_j$, $j=1,\ldots ,l$. All the costs are equal to zero and values of the other input parameters are such that the constraints they are involved in are satisfied. Introduce variables $y_{j0}=x_{j((0,1),(2,1),\bullet )}$ and $y_{j1}=x_{j((1,1),(2,1),\bullet )}$. For the constructed instance of CMCF$(F=1,T=1,c^{(rej)}_j=\infty ,p_{j,Q+1}=0)$, the following constraints must be satisfied:

$$\begin{aligned} y_{j0}+y_{j1}=d_{j1}=1,\ j=1,\ldots ,l, \end{aligned}$$

$$\begin{aligned} y_{j0},y_{j1}\in \{0,1\},\ j=1,\ldots ,l, \end{aligned}$$

$$\begin{aligned} \sum _{j=1}^l r_jy_{j0}=\sum _{j=1}^{2h} a_jy_{j0}\le U_0=A, \end{aligned}$$

$$\begin{aligned} \sum _{j=1}^l r_jy_{j1}=\sum _{j=1}^{2h} a_jy_{j1}\le U_1=A. \end{aligned}$$

It is easy to see that the constructed instance of CMCF$(F=1,T=1,c^{(rej)}_j=\infty ,p_{j,Q+1}=0)$ has a feasible solution if and only if the original instance of Partition has a solution. The relation between the two solutions is such that $j\in Y$ implies $y_{j0}=1$ and $y_{j1}=0$ and $j\in L\backslash Y$ implies $y_{j0}=0$ and $y_{j1}=1$, $j=1,\ldots ,l$. $\square$

### Theorem 4

The problem CMCF$(F=0,T=2,c^{(rej)}_j=\infty ,p_{j,Q+1}=0)$ is NP-hard even if all the costs are equal to zero, $j=1,\ldots ,n$.

### Proof

A reduction from the problem Partition is used. For any instance of Partition, construct instance of CMCF$(F=0,T=2,c^{(rej)}_j=\infty ,p_{j,Q+1}=0)$, in which $n=l$, $U_0=A$, $k_{j1}=1$, $k_{j2}=0$, $g_{j0}=g_{j1}=u_{j0}=u_{j1}=0$, $d_{j1}=0$, $d_{j2}=1$ (one container of each type *j* must be repaired on facility 0 either in time period 1 or in time period 2), and repair workforce requirements are $r_j=a_j$, $j=1,\ldots ,l$. All the costs are equal to zero and values of the other input parameters are such that the constraints they are involved in are satisfied. Introduce variables $z_{j1}=x_{j((0,1),(0,2),\bullet )}$ and $z_{j2}=x_{j((0,2),(1,2),\bullet )}$. For this instance of CMCF$(F=0,T=2,c^{(rej)}_j=\infty ,p_{j,Q+1}=0)$, the following constraints must be satisfied:

$$\begin{aligned} z_{j1}+z_{j2}=d_{j2}=1,\ j=1,\ldots ,l, \end{aligned}$$

$$\begin{aligned} z_{j1},z_{j2}\in \{0,1\},\ j=1,\ldots ,l, \end{aligned}$$

$$\begin{aligned} \sum _{j=1}^l r_jz_{j1}=\sum _{j=1}^l a_jz_{j1}\le U_0=A, \end{aligned}$$

$$\begin{aligned} \sum _{j=1}^l r_jz_{j2}=\sum _{j=1}^l a_jz_{j2}\le U_0=A. \end{aligned}$$

It is easy to see that the described instance of CMCF$(F=0,T=2,c^{(rej)}_j=\infty ,p_{j,Q+1}=0)$ has a solution if and only if the original instance of Partition has a solution. The relation between the two solutions is such that $j\in Y$ implies $z_{j1}=1$ and $z_{j2}=0$ and $j\in L\backslash Y$ implies $z_{j1}=0$ and $z_{j2}=1$, $j=1,\ldots ,l$.

$$\square $$

Theorems 1–4 set the borderline between easy and hard cases of the problem CMCF with no rejection allowed: The problem is polynomially solvable if $F=0$, $T=1$ and all containers are repairable, and it is NP-hard if some containers are not repairable or one of the parameters *F* and *T* is increased by one.

We conclude this section with a remark on an equivalence of the problem CMCF with no rejection allowed and a problem with high rejection costs. Calculate an upper bound, denoted as *B*, on the total cost of a feasible solution of the problem CMCF obtained if every arriving container is supposed to be accepted, inspected, transported, repaired and stored:

27 $$\begin{aligned}&B=\min \{B_1,B_2\},\hbox { where}\nonumber \\&B_1=\sum _{u=1}^n\sum _{t=1}^T k_{ut}\Big (c^{(ins)}_u +\max _{1\le q\le Q}\Big \{c^{(rep)}_{uq}\Big \} +\max _{1\le f\le F}\Big \{c^{(tra)}_f+(T-t)c^{(hol)}_f\Big \}\Big ), \nonumber \\&B_2=\sum _{t=1}^T\Big [m\cdot \max _{1\le f\le F}\Big \{c^{(tra)}_f\Big \}+\sum _{f=0}^F \Big (V_f+U_f+(T-t)W_fc^{(hol)}_f\Big ). \end{aligned}$$

### Remark 1

The problem CMCF with rejection costs $c^{(rej)}_j=B$, $j=1,\ldots ,n$, has an optimal solution with the total cost not exceeding $B-1$ if and only if it has a feasible solution with no rejected container.

## Computer experiments

Computer experiments aim to demonstrate that the instances with the practical input data can be solved in an acceptable time, to find input parameters that affect applicability of the relaxed problem solution, to see how the solution time and the share of the rejected containers in an optimal solution depends on the rejection costs, and to detect the most difficult benchmark instances. In the experiments, 30 series of random instances are constructed. The number of container types is $n=4$ and the number of facilities is three ($F=2$) in any instance. Each series is specified by a triple $(T,Q,c^{(rej)})$, where $c^{(rej)}$ denotes a common single container rejection cost. We consider $T\in \{7,14,30\}$, $Q\in \{5,9\}$, $c^{(rej)}\in \{100,200,300,500,B\}$, where *B* is the big number defined in (27). Notation $c^{(rej)}=c$ means that $c^{(rej)}_j=c$ for all $j=1,\ldots ,n$. For each series, 20 random instances of the problem CMCF are generated. For each instance of the same series, the following within the intervals of equally distributed input data are randomly generated:

- number of containers of type *j* arriving to facility 0 at the beginning of time period *t*: $k_{jt}\in [100,300]$, $j=1,\ldots ,n$, $t=1,\ldots ,T$;
- initial inventory of non-inspected containers of type *j* at facility *f* at the beginning of time period 1: $u_{jf}\in [0,100]$, $j=1,\ldots ,n$, $f=0,1,\ldots ,F$;
- initial inventory of good quality containers of type *j* at facility *f* at the beginning of time period 1: $g_{jf}\in [0,100]$, $j=1,\ldots ,n$, $f=0,1,\ldots ,F$;
- demand of containers of type *j* at the end of period *t*: $d_{jt}\in [\lfloor 0.8D_j\rfloor ,\lfloor 1.2D_j\rfloor ]$, where $D_j=\frac{\sum _{f=0}^F (u_{jf}+g_{jf})+\sum _{\tau =1}^T k_{j\tau }}{T}$ is the average amount of available containers per time period, $j=1,\ldots ,n$, $t=1,\ldots ,T$;
- transportation capacity: $m\in [\lfloor 0.8M\rfloor ,\lfloor 1.2M\rfloor ]$, where $M=\frac{F\sum _{j=1}^nD_j}{F+1}$ is the average amount of available containers per time period multiplied by $\frac{F}{F+1}$ to account for the containers which are not moved from facility 0;
- nominator in the probability that the original quality level of any single inspected container of type *j* is *q*: $p_{j0}\in [5,10]$, $p_{j,Q+1}\in [0,5]$ and $p_{jq}=\Big \lfloor \frac{(100-p_{j0}-p_{j,Q+1})\ x_q}{\sum _{h=1}^Q x_h}\Big \rfloor$, where random numbers $x_q\in [0,100]$ for $q\in \{1,\ldots ,Q\}$, $j=1,\ldots ,n$. For each *j*, calculate $\Delta _j=100-\sum _{q=0}^{Q+1}p_{jq}$. Note that $\Delta _j\le Q$ due to the rounding down operation for $p_{jq}$. Introduce set of indices $H=\{1,\ldots ,Q\}$. For each *j*, generate random number $h\in H$ and re-set $p_{jh}:=p_{jh}+1$. Remove *h* from *H* and repeat generation of *h* and re-setting of $p_{jh}$. Repeat this process $\Delta _j$ times to get $\sum _{q=0}^{Q+1}p_{jq}=100$, $j=1,\ldots ,n$;
- number of working-hours required to inspect one container of type *j* at any facility: $s_j=\frac{s'_j}{60}$, where $s'_j\in [20,60]$, $j=1,\ldots ,n$;
- upper bound on the available working-hours for inspection in each time period at facility *f*: $V_f\in [\lfloor 1.2I\rfloor ,\lfloor 1.8I\rfloor ]$, where $I=\frac{\sum _{j=1}^ns_jD_j}{F+1}$ is the average amount of working-hours required to inspect all the available containers per time period and facility, $f=0,1,\ldots ,F$;
- number of working-hours required to repair one container of type *j* and quality level $q\in \{1,\ldots ,Q\}$ at any facility: $r_{jq}=\frac{r'_{jq}}{60}$, where $r'_{jq}\in [20q,20(q+1)]$, $j=1,\ldots ,n$, $q=1,\ldots ,Q$;
- upper bound on working-hours available for repair work in any time period at facility *f*: $U_f\in [\lfloor 1.2J\rfloor ,\lfloor 1.8J\rfloor ]$, where $J=\frac{\sum _{j=1}^n\sum _{q=1}^Qp_{jq}r_{jq}D_j}{100(F+1)}$ is the average amount of working-hours required to repair all the available containers of quality levels $1,\ldots ,Q$ per time period and facility, $f=0,1,\ldots ,F$;
- storage capacity of facility *f*: $W_f\in [\lfloor 0.9\frac{(3\sum _{j=1}^nD_j)}{F+1}\rfloor ,\lfloor 1.1\frac{(3\sum _{j=1}^nD_j)}{F+1}\rfloor ]$, where $\frac{3\sum _{j=1}^nD_j}{F+1}$ is the tripled average amount of the available containers per time period and facility to allow storage of the containers from the previous time periods, $f=0,1,\ldots ,F$;
- cost of inspection of one container of type *j* and any quality level at any facility: $c^{(ins)}_j=20s_j$, where 20 is the cost of one working-hour of inspection work, $j=1,\ldots ,n$.
- cost of repair of one container of type *j* and quality level *q* at any facility: $c^{(rep)}_{jq}=30r_{jq}$, where 30 is the cost of one working-hour of repair work, $j=1,\ldots ,n$, $q=1,\ldots ,Q$, $c^{(rep)}_{j0}=0$ (for good quality containers);
- cost of transportation of one container of any type from facility 0 to facility *f*: $c^{(tra)}_f\in [50+10f,150+10f]$ , $f=1,\ldots ,F$;
- holding cost of one container of any type at facility *f* when passing from any time period *t* to time period $t+1$, $t=0,1,\ldots ,T$: $c^{(hol)}_f\in [1+\frac{F-f}{2},2+\frac{F-f}{2}]$, $f=0,1,\ldots ,F$.

The values of the input parameters are generated to correlate with values of these parameters that we observed in a practical environment, except for the rejection cost $c^{(rej)}\in \{100,200,300,500,B\}$ whose range was determined experimentally in order to demonstrate dependence of solution times and other solution characteristics of the rejection cost. In particular, $n=4$ container types are standard 20-foot and 40-foot containers and 20-foot and 40-foot reefer (refrigerated) containers. Three facilities ($F=2$) are an inspection and repair shop at the container terminal and two other repair shops, a little remote. The number of time periods $T\in \{7,14,30\}$ correspond to the one-week, two-week and one-month planning horizon. The number of quality levels $Q\in \{5,9\}$ is obtained by classifying damage types with similar characteristics into smaller ($Q=5$) or larger ($Q=9$) number of groups.

The random instances of CMCF and CMCF-R were solved with Python API of CPLEX 12.10 on a MacBook Pro with Intel Core i7 2.6 GHz processor and 16 GB of RAM under macOS Big Sur. Each instance of CMCF-R was solved in less than 1 second. The CPU time of CPLEX for CMCF was limited to three hours for each instance and the optimality gap (guaranteed solution relative error) was set to zero. Tables 2, 3, 4, 5 and 6 contain the following information for each series $(T,Q,c^{(rej)})$:

- number of instances for which no feasible solution was found within the time limit (row $\#_{infeas}$). For any instance in our experiments, either both problems CMCF and CMCF-R had feasible solutions or both of them had no feasible solution, which was established by running CPLEX until a feasible solution was found or infeasibility was established. Since any instance of CMCF-R was solved in less than 1 second, parameter $\#_{infeas}$ is independent of the solution time limit;
- number of instances of CMCF for which an optimal solution was provably found within the time limit (row $\#_{opt}$). Note that the number of feasible instances for which no optimal solution was provably found within the time limit is equal to $20-\#_{infeas}-\#_{opt}$;
- average and maximal run time of CPLEX for CMCF in minutes to find a solution with proven optimality (rows “Mean time to opt” and “Max time to opt,” respectively);
- mean ratio of the number of rejected containers to the number of all arriving containers over feasible instances (row “Mean $\frac{{\#\ \hbox {rejected}}}{\#\ \hbox {all}}$”), where “$\#\ \hbox {all}$”$=\sum _{j=1}^n\sum _{t=1}^T k_{jt}$ .

**Table 2:** Computer experiments for $c^{(rej)}=100$

(*T*, *Q*)	(7,5)	(7,9)	(14,5)	(14,9)	(30,5)	(30,9)
$\#_{infeas}$	0	1	0	0	0	0
Time limit *k*=5min						
$\#_{opt}$	10	10	0	0	0	0
Mean time to opt, min	$<1$	1	–	–	–	–
Max time to opt, min	$<1$	2	–	–	–	–
Mean $\frac{\#\ \hbox {rejected}}{\#\ \hbox {all}}$	0.128	0.154	0.244	0.146	0.217	0.201

Time limit *k*=1h						
$\#_{opt}$	12	13	0	0	0	0
Mean time to opt, min	3	5	–	–	–	–
Max time to opt, min	31	39	–	–	–	–
Mean $\frac{\#\ \hbox {rejected}}{\#\ \hbox {all}}$	0.092	0.107	0.244	0.146	0.217	0.201

Time limit *k*=2h						
$\#_{opt}$	13	13	0	0	0	0
Mean time to opt, min	8	5	–	–	–	–
Max time to opt, min	66	39	–	–	–	–
Mean $\frac{\#\ \hbox {rejected}}{\#\ \hbox {all}}$	0.093	0.076	0.244	0.146	0.217	0.201

Time limit *k*=3h						
$\#_{opt}$	13	13	0	0	0	0
Mean time to opt, min	8	5	–	–	–	–
Max time to opt, min	66	39	–	–	–	–
Mean $\frac{\#\ \hbox {rejected}}{\#\ \hbox {all}}$	0.091	0.076	0.244	0.146	0.217	0.201

**Table 3:** Computer experiments for $c^{(rej)}=200$

(*T*, *Q*)	(7,5)	(7,9)	(14,5)	(14,9)	(30,5)	(30,9)
$\#_{infeas}$	0	0	0	0	0	0
Time limit *k*=5min						
$\#_{opt}$	8	7	0	0	0	0
Mean time to opt, min	2	1	–	–	–	–
Max time to opt, min	5	2	–	–	–	–
Mean $\frac{\#\ \hbox {rejected}}{\#\ \hbox {all}}$	0.001	0	0	0.001	0.001	0.002

Time limit *k*=1h						
$\#_{opt}$	11	8	0	0	0	0
Mean time to opt, min	8	6	–	–	–	–
Max time to opt, min	56	43	–	–	–	–
Mean $\frac{\#\ \hbox {rejected}}{\#\ \hbox {all}}$	0.001	0	0	0.001	0.001	0.001

Time limit *k*=2h						
$\#_{opt}$	13	9	0	0	0	0
Mean time to opt, min	8	19	–	–	–	–
Max time to opt, min	56	118	–	–	–	–
Mean $\frac{\#\ \hbox {rejected}}{\#\ \hbox {all}}$	0.001	0	0	0.001	0.001	0.001

Time limit *k*=3h						
$\#_{opt}$	13	9	0	0	0	0
Mean time to opt, min	27	19	–	–	–	–
Max time to opt, min	131	118	–	–	–	–
Mean $\frac{\#\ \hbox {rejected}}{\#\ \hbox {all}}$	0.001	0	0	0.001	0	0.001

**Table 4:** Computer experiments for $c^{(rej)}=300$

(*T*, *Q*)	(7,5)	(7,9)	(14,5)	(14,9)	(30,5)	(30,9)
$\#_{infeas}$	0	1	0	0	0	0
Time limit *k*=5min						
$\#_{opt}$	5	7	0	0	0	0
Mean time to opt, min	2	1	–	–	–	–
Max time to opt, min	4	3	–	–	–	–
Mean $\frac{\#\ \hbox {rejected}}{\#\ \hbox {all}}$	0	0	0	0.001	0.003	0.001

Time limit *k*=1h						
$\#_{opt}$	7	10	0	0	0	0
Mean time to opt, min	11	10	–	–	–	–
Max time to opt, min	42	44	–	–	–	–
Mean $\frac{\#\ \hbox {rejected}}{\#\ \hbox {all}}$	0	0	0	0.001	0.003	0.001

Time limit *k*=2h						
$\#_{opt}$	9	11	0	0	0	0
Mean time to opt, min	32	17	–	–	–	–
Max time to opt, min	117	88	–	–	–	–
Mean $\frac{\#\ \hbox {rejected}}{\#\ \hbox {all}}$	0	0	0	0.001	0.003	0.001

Time limit *k*=3h						
$\#_{opt}$	9	11	0	0	0	0
Mean time to opt, min	32	17	–	–	–	–
Max time to opt, min	117	88	–	–	–	–
Mean $\frac{\#\ \hbox {rejected}}{\#\ \hbox {all}}$	0	0	0	0.001	0.003	0.001

**Table 5:** Computer experiments for $c^{(rej)}=500$.

(*T*, *Q*)	(7,5)	(7,9)	(14,5)	(14,9)	(30,5)	(30,9)
$\#_{infeas}$	1	1	0	0	0	0
Time limit *k*=5min						
$\#_{opt}$	13	3	0	0	0	0
Mean time to opt, min	1	2	–	–	–	–
Max time to opt, min	4	4	–	–	–	–
Mean $\frac{\#\ \hbox {rejected}}{\#\ \hbox {all}}$	0	0	0	0.000833	0	0.002985

Time limit *k*=1h						
$\#_{opt}$	14	8	0	0	0	0
Mean time to opt, min	2	12	–	–	–	–
Max time to opt, min	12	34	–	–	–	–
Mean $\frac{\#\ \hbox {rejected}}{\#\ \hbox {all}}$	0	0	0	0.001	0	0.003

Time limit *k*=2h						
$\#_{opt}$	14	8	0	0	0	0
Mean time to opt, min	2	12	–	–	–	–
Max time to opt, min	12	34	–	–	–	–
Mean $\frac{\#\ \hbox {rejected}}{\#\ \hbox {all}}$	0	0	0	0.001	0	0.003

Time limit *k*=3h						
$\#_{opt}$	14	8	0	0	0	0
Mean time to opt, min	2	12	–	–	–	–
Max time to opt, min	12	34	–	–	–	–
Mean $\frac{\#\ \hbox {rejected}}{\#\ \hbox {all}}$	0	0	0	0.001	0	0.003

**Table 6:** Computer experiments for $c^{(rej)}=B$.

(*T*, *Q*)	(7,5)	(7,9)	(14,5)	(14,9)	(30,5)	(30,9)
$\#_{infeas}$	0	0	0	0	0	1
Time limit *k*=5min						
$\#_{opt}$	6	5	0	0	0	0
Mean time to opt, min	1	1	–	–	–	–
Max time to opt, min	3	4	–	–	–	–
Mean $\frac{\#\ \hbox {rejected}}{\#\ \hbox {all}}$	0	0	0	0	0	0.002

Time limit *k*=1h						
$\#_{opt}$	8	12	0	0	0	0
Mean time to opt, min	4	15	–	–	–	–
Max time to opt, min	15	58	–	–	–	–
Mean $\frac{\#\ \hbox {rejected}}{\#\ \hbox {all}}$	0	0	0	0	0	0.002

Time limit *k*=2h						
$\#_{opt}$	8	12	0	0	0	0
Mean time to opt, min	4	15	–	–	–	–
Max time to opt, min	15	58	–	–	–	–
Mean $\frac{\#\ \hbox {rejected}}{\#\ \hbox {all}}$	0.0002	0	0	0	0	0.002

Time limit *k*=3h						
$\#_{opt}$	8	14	0	0	0	0
Mean time to opt, min	4	31	–	–	–	–
Max time to opt, min	15	130	–	–	–	–
Mean $\frac{\#\ \hbox {rejected}}{\#\ \hbox {all}}$	0	0	0	0	0	0.002

For each instance we denote by $C^{(R)}$ the value of an optimal solution of this instance of CMCF-R, and by $C^{(k)}$ the value of a best solution of that instance of CMCF found by CPLEX in time *k*, $k\in \{5\hbox {min},1\hbox {h},2\hbox {h},3\hbox {h}\}$. The mean and maximal CPLEX optimality gaps and the mean and maximal ratios $\frac{C^{(k)}}{C^{(R)}}$, $k\in \{5\hbox {min},1\hbox {h},2\hbox {h},3\hbox {h}\}$, over feasible instances were less than 0.5% for all series but the maximal gaps and the maximal ratios for the series with $c^{(rej)}=B$. For the series with $c^{(rej)}=B$ the maximal gaps did not exceed 3% and the maximal ratios $\frac{C^{(k)}}{C^{(R)}}$ reached 32.5%.

If the ratio of the integer to the non-integer objective value is small, then rounding of non-integer variables in the solution of the relaxed problem may lead to an acceptable solution of the original integer problem. However, for instances with high rejection cost where this ratio is large, the rounding approach may lead to a bad solution of the original problem. An explanation is that even a small part of the rejected container quantity is costly if $c^{(rej)}$ is large. Thus, if the rejection costs are relatively small and a fast solution of CMCF is needed, then the relaxed problem can be solved and its solution can be converted into an applicable solution of the original problem.

The experimental results are acceptable. For a practical mid-term planning problem the solver can be run the whole night (about 12 hours). If there is a single input data scenario, then 12 hours can be considered as the solution time limit for solving one instance. If there are *k* scenarios, then 12/*k* can be taken as the time limit for each scenario.

The average ratio of the rejected containers to all containers, $\frac{\#\ \hbox {rejected}}{\#\ \hbox {all}}$, decreases as the rejection cost $c^{(rej)}$ increases. A significant drop of this ratio is observed between $c^{(rej)}=100$, where the ratio reaches 24.4%, and $c^{(rej)}=200$, where it drops to 0.2%. For $c^{(rej)}\ge 200$, the ratio does not exceed 0.3%. This observation can be used to accelerate the solution process for the hard instances with no container rejection or large rejection costs: such an instance can be solved with a modified smaller rejection cost, and if the number of rejected containers is acceptable, then the solution of the modified instance is acceptable for the original instance as well.

Several experiments were conducted to find hard benchmark instances of CMCF. Two such instances with $(T,Q,c^{(rej)})=(360,5,B)$ were detected. The gap values and the ratios $\frac{C^{(kh)}}{C^{(R)}}$, $k=1,\ldots ,10$, for these instances are given in Table 7. Not surprisingly, they decrease with increasing run time. However, the optimality gaps for both instances remain more than 22% and 28% and the ratios $\frac{C^{(kh)}}{C^{(R)}}$ remain more than 1.29 and 1.45 after ten hours of running CPLEX. All data, including two hard instances can be downloaded from Kovalyov et al. 2022 after this paper has been accepted for publication.

**Table 7:** Hard benchmark instances, $(n,F,T,Q,c^{(rej)})=(4,2,360,5,B)$

*k* (hours)	1	2	3	4	5	6	7	8	9	10
Instance 1										
Gap	0.428	0.337	0.297	0.282	0.276	0.271	0.266	0.262	0.246	0.226
$\frac{C^{(kh)}}{C^{(R)}}$	1.753	1.514	1.428	1.398	1.386	1.376	1.368	1.36	1.33	1.297
Instance 2										
Gap	0.516	0.455	0.431	0.42	0.401	0.39	0.382	0.378	0.33	0.287
$\frac{C^{(kh)}}{C^{(R)}}$	2.141	1.9	1.822	1.786	1.73	1.697	1.675	1.665	1.548	1.453

## Conclusions

We introduce and model a tactical cost minimizing resource planning problem for inspection and repair of empty containers at a container terminal. The containers are of *n* types and different quality levels, which define their repair costs. The objective function includes the total rejection, inspection, repair, transportation and holding costs. A time-dependent integer linear constrained min-cost multi-commodity network-flow formulation, denoted as problem CMCF, is proposed. Problem CMCF is shown to be solvable in *O*(*n*) time if there is a single facility, a single time period, container rejection is not allowed and all containers are repairable. If there exist non-repairable containers of each type, there is one facility ($F=0$) and one time period ($T=1$), or there are at least two facilities, or at least two time periods, then the problem is NP-hard. Computer experiments with randomly generated instances demonstrate that all the instances with 3 facilities ($F=2$), 4 container types, up to 9 container quality levels and up to 30 time periods are solved with an optimality gap of less than 3% in 5 minutes. Benchmark instances are found with 3 facilities, 4 container types, 5 container quality levels and 360 time periods, for which an optimality gap of more than 28% remains even after ten hours of running CPLEX. Our research is of an initiative nature in relations with an industrial partner. If there will be a commercial interest, then the developed software can be included in the port logistics information system and tuned for a good performance in a real-life environment.

For future research, it is interesting to study optimal planning problems of freight container inspection and repair with other real-life assumptions, objectives and constraints. Handling uncertainty and on-line nature of these problems is of utmost importance. Developing efficient meta-heuristic approaches to solve these problems can be an alternative to using MILP solvers.
